# Rationale and design of a randomized clinical trial of integrated eHealth for PrEP and medications for opioid use disorders for women in the criminal legal system. The Athena study

**DOI:** 10.1186/s13722-024-00534-x

**Published:** 2025-01-17

**Authors:** Jaimie P. Meyer, Stacey Brunson, Carolina R. Price, Morgan Mulrain, Julie Nguyen, Frederick L. Altice, Tassos C. Kyriakides, Karen Cropsey, Ellen Eaton

**Affiliations:** 1 Yale School of Medicine, Section of Infectious Diseases, New Haven, Connecticut, USA; 2 Yale School of Public Health, Chronic Disease Epidemiology, New Haven, Connecticut, USA; 3 University of Alabama at Birmingham School of Medicine, Birmingham, AL USA; 4 Yale School of Public Health, Epidemiology of Microbial Diseases, New Haven, Connecticut, USA; 5 Yale Center for Analytical Sciences, Yale School of Public Health, New Haven, Connecticut, USA; 6 135 College Street, Suite 323, New Haven, CT 06510 USA

**Keywords:** HIV prevention, Women involved in criminal legal systems (WICL), Pre-exposure prophylaxis (PrEP), Medications for opioid use disorder (MOUD), Opioid use disorder (OUD)

## Abstract

**Background:**

Women involved in the criminal legal system have elevated rates of opioid use disorder, which is treatable, and HIV, which is preventable with pre-exposure prophylaxis (PrEP). There are significant social and structural barriers to integrated delivery of PrEP and medications for opioid use disorder (MOUD), limiting women’s ability to access these life-saving interventions. In a two parallel-arm randomized controlled trial, we are assessing an innovative eHealth delivery model that integrates PrEP with MOUD and is tailored to meet the specific needs of women involved in the criminal legal system.

**Methods:**

We will recruit and enroll 250 women involved in the criminal legal system with opioid use disorder across two diverse settings (New Haven, CT and Birmingham, AL). Participants will be randomized to (a) the “Athena strategy,” which includes a PrEP decision aid and integrated PrEP/MOUD delivery via eHealth; or (b) enhanced standard of care (SOC) that includes a decision aid-only. During 6-month follow-up, we will assess PrEP initiation as the primary clinical outcome and implementation outcomes that include acceptability, adoption, feasibility, fidelity, implementation cost, and sustainability.

**Discussion:**

Results could help determine if reducing the social and structural barriers to PrEP and MOUD for women involved in the criminal legal system will facilitate engagement in treatment and prevention services, thus alleviating health disparities.

**Trial registration:**

Clinicaltrials.gov (NCT05547048). Registered September 15, 2022. https://clinicaltrials.gov/study/NCT05547048?term=NCT05547048&rank=1.

**Supplementary Information:**

The online version contains supplementary material available at 10.1186/s13722-024-00534-x.

## Background

The U.S. incarcerates more women in closed detention settings (i.e., prisons and jails) than any other country worldwide [1], though most criminal legal system-involvement is through community-based supervision that includes jail-diversion programs, probation, and parole [2]. Women involved in criminal legal systems (WICL) have elevated rates of untreated opioid use disorder (OUD) and HIV [3–5], are twice as likely as incarcerated men to have HIV [6, 7], and have higher rates of comorbid Hepatitis C and psychiatric and substance use disorders (SUD) compared to community-based women and incarcerated men [8]. For WICL, HIV risk is largely attributable to overlapping interpersonal networks involving transactional sex, injection drug and other substance use, unstable housing, and social marginalization [9, 10]. Women in high-risk networks and relationships may be exposed to intimate partner violence (IPV) that reduces their autonomy to modify drug use behaviors or engage in health-promoting activities [11–13]. Because of their substantial risk for HIV [14], WICL are a priority population for HIV prevention as part of national and global strategies to Ending the HIV Epidemic [15, 16]. 

HIV pre-exposure prophylaxis (PrEP) reduces HIV transmission to women and is partner- and event-independent [17–19], which is empowering for WICL who may rely on partners for basic subsistence and have limited social capital to negotiate male partners’ condom use [20–22]. Despite demonstrated efficacy in clinical trials, PrEP use in the U.S. remains low overall and especially low in women. Less than 8.5% of clinically eligible women have received tenofovir disoproxil fumarate/emtricitabine (TDF/FTC) since it was FDA-approved for PrEP in 2012 and tenofovir alafenamide fumarate/emtricitabine (TAF/FTC) is still not approved for people whose HIV risk is from vaginal sex [23–25]. PrEP is under-utilized due to provider biases, patients’ low risk perception, structural factors, including stigma in healthcare settings, and competing demands.

PrEP adoption gaps are significant for WICL. To access PrEP, women must accurately estimate their own and their partners’ HIV risk and be aware of PrEP, which are major obstacles for WICL and women who use drugs [26–31]. Patient-centered decision aids can align risk perceptions and increase PrEP awareness by merging patient preferences with evidence-based practices. In a prior study, we applied international standardized criteria to develop, test, and report on the first PrEP decision aid [32] and tailored it to women with OUD [33, 34], including trauma-responsive adaptations [35]. We found the decision aid was feasible to administer, acceptable to women and stakeholders, and effective at modifying PrEP preference, but had modest effect on PrEP uptake, in part, because women need to be actively linked to HIV prevention services. We also have successfully enrolled > 100 WICL and their risk network members in an eHealth program for PrEP, demonstrating feasibility, acceptability, and efficacy at increasing PrEP initiation, though the program did not address MOUD [36, 37]. This study builds on our prior work by integrating MOUD and PrEP into an eHealth intervention.

This study responds to the urgent need to reach WICL for life saving, evidence-based HIV PrEP and MOUD by using an innovative healthcare delivery model that integrates services and lowers social and structural barriers to entry. We will reach WICL and evaluate key clinical and implementation outcomes of an integrated PrEP/MOUD delivery model using eHealth and combined with a dedicated PrEP decision aid, as compared to the PrEP decision aid alone with standard of care. We aim to generate sufficient data to drive innovative solutions into the next phase of scaling up and implementing the eHealth delivery model for integrated PrEP/MOUD.

## Methods

### Study design

This study is a multi-site two parallel-arm unblinded randomized clinical trial comparing the “Athena strategy,” a low-demand model that includes a PrEP decision aid and eHealth delivery of integrated PrEP/MOUD, to a PrEP decision aid-only in terms of patient-level engagement in the PrEP care continuum among WICL with OUD, considering key participating site differences. The primary clinical outcome is PrEP initiation over six months; secondary outcomes are 6-month PrEP retention and engagement in the OUD treatment cascade. We will assess for the complexity of SUD treatment (e.g., court-mandated vs. voluntary; behavioral vs. MOUD) and changes in treatment engagement over time. Exploratory outcomes include diagnosis and treatment of comorbid Hepatitis C and sexually transmitted infections. We will also assess scale-up potential of the Athena strategy in terms of modelled long-term outcomes and how stakeholders interact with eHealth for integrated PrEP/MOUD in WICL in two diverse epidemiological and implementation contexts, using standardized definitions of implementation outcomes [38]. In a state transition model, we will estimate averted HIV infections and quality-adjusted life-year (QALY) gains at individual and population levels over a 10-year time horizon [39]. We will incorporate multi-level perspectives on implementation through focus groups using nominal group technique (NGT), structured surveys, and in-depth interviews.

### Study setting

Participants will be recruited at two sites: New Haven, CT and Birmingham, AL, which are two diverse settings with distinct epidemiological and implementation contexts. Both states share a common need for PrEP scale-up, and AL is a designated high priority state for Ending the HIV Epidemic (EHE) [15]. Additionally, CT and AL have different HIV and opioid micro-epidemics, resources, and criminal legal systems, allowing us to examine the full continuum of PrEP delivery issues for WICL.

### Recruitment

We will recruit participants from criminal legal sites and other community settings that serve WICL with OUD. Study participants will be recruited from advertisements placed in probation and parole offices, “community corrections” programs (as applicable), courts, halfway houses or transitional housing programs, area health centers, outpatient drug treatment programs, within prisons/jails, and through social media. At the AL site, additional recruitment activities focus on hospitalized patients (pre-screened for age, gender, and opioid use disorder). Trained research assistants will be onsite at each of the community sites 1–2 days per week during the recruitment phase to inform potential participants about the project. We will also accept self-, peer-, and provider- referrals. Interested individuals can self-refer using a QR code to a HIPAA-secure online Qualtrics link, through mechanisms we already have in place with multiple prior and ongoing studies. The referral link will only contain basic contact information, and state that they agree to be contacted and safest/preferred method of contact. Interested individuals will also be able to self-refer through a private protected phone line to our trained research assistant. Service providers may refer potential participants through the QR code to a HIPAA-secure online Qualtrics link, after they have obtained permission from the potential participant to forward their name and contact information for the study. The referral link will only contain basic contact information. We will not ask probation or parole officers (or others in charge of CL supervision) to directly refer participants to avoid any real or perceived coercion.

For potentially interested individuals who are incarcerated at the time of recruitment, we will complete recruitment and screening in the following way: Once appropriate approvals are obtained from CL sites and if research staff are approved to enter the facilities, we will interact directly with potentially interested participants to introduce the study, answer any initial questions, conduct brief eligibility screening, and provide contact information to follow up with the research teams following release. With appropriate approvals from CL sites, we will distribute study promotional material in the facilities through the discharge planners so that women can have our contact information and call our research site through a dedicated private phone line. Women recruited from prisons or jails may complete eligibility screening over the phone while incarcerated but will not complete any study-related HIV screening nor be formally offered enrollment until after returning to communities.

### Eligibility criteria

Research assistants will contact all interested individuals to complete a more detailed study eligibility screen in REDCap. Referred clients will be assessed for the following study eligibility criteria: (1) cis-women; (2) ages 18-59y (because they experience the highest HIV risk); (3) have access to a working mobile or landline phone; (4) CL-involved (currently on probation, parole, intensive pretrial or community supervision, or are within 12 months after release from prison/jail;) (5) meet clinical criteria for PrEP; and (6) have OUD (regardless of baseline MOUD treatment status), as determined by a SAMHSA-recommended single-item screening question: *How many times in the past year have you used an illegal drug or a prescription medication for nonmedical reasons?* and followed by confirmation of OUD using the validated Rapid Opioid Dependence Screen (RODS) [40]. 

Referred clients will be excluded if they are: (1) unable or unwilling to provide informed consent; (2) pregnant or breast-feeding; (3) currently taking PrEP at the time of study enrollment; (4) not comfortable conversing in English or Spanish; or (5) test positive for HIV. There is no exclusion based on health or technology literacy, homelessness, trauma, substance use or psychiatric disorders, and we will address these factors in our intervention and analysis.

As part of screening for study eligibility, individuals will undergo rapid point of care screening for HIV with the ORAQUICK Advance^®^ Rapid HIV-1/2 Antibody Test. Participants who screen negative meet inclusion criteria. Participants who have an indeterminate or positive result for either will be sent for confirmatory testing and referred for HIV care if confirmed positive. Potential participants will also be screened for pregnancy with a urine hCG. Participants testing HIV + or pregnant will be counseled, referred for care, and excluded from the study.

We will verify CL involvement using publicly available websites and by directly contacting supervising officers with a signed release of information; all participants recruited from “community corrections,” parole, probation, or closed detention settings are already known to be CL-involved. Of note, we will only enroll women who are residing in the community. Women who are informed about the study during incarceration and interested in participating can enroll following return to communities; no one will be enrolled while they are incarcerated.

Those who meet all inclusion criteria will be informed about the study, offered enrollment, and meet with our trained research assistant either onsite immediately or at a scheduled appointment convenient for the participant. At this visit, participants will be formally enrolled by signing a compound authorization/consent to participate (that includes a specific informed consent for telehealth, focus groups, and PrEP if eligible) and a release of information from specified sources for medical, psychiatric, social, and CL system data. Electronic consenting will be done using REDCap. Participants will be sent a PDF of the IRB-approved Compound Authorization Form to their preferred email or text to follow along with the interviewer as the information is displayed on the computer screen. We will perform teach-back techniques to ensure clarity of the informed consent. Participants will indicate consent with a digital signature. Interviewers will also electronically sign a copy of the consent form indicating that all information has been reviewed and understood by the participant. REDCap will save a time-stamped copy of both signed consent forms; the participant will also be offered a copy of the consent form.

Key stakeholders will also be invited to participate in the study. Inclusion criteria for stakeholders are: (1) adults ≥ 18 years old; (2) serve on the community advisory board at either site. Exclusion criteria for this group are: (1) unable or unwilling to provide informed consent; or (2) not comfortable conversing in English.

### Method of Assignment/Randomization

We will use the REDCap automated randomization tool to assign participants into one of two study arms 1:1 and stratified by: (1) site (AL or CT) to balance arms by potentially unmeasured factors that differ by site; (2) past 6-month stimulant use, given high prevalence of stimulant use in this population and association of stimulant use with reduced MOUD retention; [41] and (3) receipt of MOUD at baseline. We selected these strata because we hypothesize these are important effect modifiers and that the implementation strategy will differentially affect these groups. Randomization will be irrespective of baseline decisional preference for PrEP because we expect nearly all will have high interest in PrEP after the decision aid (80–90% based on our pilot clinical trial) [34], but if there are differences in decisional preference between the two groups, we will assess this in the analysis.

#### Intervention arm

Participants randomized to the Athena strategy arm will receive the decision aid and integrated PrEP/MOUD via eHealth- a low-demand model designed to reduce social and structural barriers to entry. The research team will schedule an appointment for an eHealth visit and provide instructions on how to access and initiate the visit. Acknowledging high rates of homelessness and IPV-exposure and limited health literacy, we will use teach-back methods to ensure participants can access eHealth safely. At the planned appointment time, the participant will check-in using the dedicated electronic health record (EHR) at each site via mobile app or website. Each EHR provides appointment reminders, clinician interaction, prescription refills, and lab orders and results. The check-in will generate an encounter so that the patient can be “arrived” by the virtual front desk. eHealth will be delivered by the onsite PrEP clinician, who will operate separately from the investigative team to minimize potential bias. Clinicians for this study are both advanced practice providers (APRN or PA) with active licenses to practice medicine in the relevant state (CT or AL), have experience providing PrEP and MOUD, and have experience working with the target population of WICL. The clinicians for this study will not contribute to the evaluation or assessment of outcomes to minimize potential bias. Clinicians are based in a community site and can include community-based PrEP navigators/case managers via videoconferencing if needed, who can help address potential structural barriers (e.g., insurance, copay coverage) to accessing PrEP through eHealth. We selected each of these community sites because both are sites for clinical care and clinical research that serve large populations who are involved with the criminal legal system. Unlike the current requirements for billing reimbursement, eHealth allows patients to be at home (to reduce care burden for WICL) rather than being seen at an established medical site; they may also choose an in-person interaction at the community site. The reason for this flexibility is to ensure that care can be delivered, regardless of technology access limitations, and mechanism of care delivery is one of the things we propose to measure as part of care delivery. For example, if a participant has limited broadband access or limited phone minutes, they may be unable to participate in a telehealth visit and may opt instead to come into our community site and use a study iPad/desktop to meet virtually with the Athena PrEP clinician, or to meet with the Athena PrEP clinician in person.

Prior to the visit, the clinician will complete a pre-visit checklist that includes reviewing output from the decision aid and point-of-care (POC) testing in REDCap, reviewing the electronic medical record, and ensuring the telehealth visit is appropriately scheduled. As part of the pre-visit activities, the Athena clinician will review and enter participant-entered and POC baseline assessment data into the EHR, using a preset template with Smart-phrases to facilitate provider communication across health systems. During the encounter, clinicians will discuss the EHR documentation described above with the patient, videoconference (synchronously) with participants and write a note in the EHR, based on a stored template. The clinician will follow standard clinical guidelines for PrEP and discuss patient preference [42]. If clinically indicated and preferred, the clinician will electronically prescribe PrEP to the patient’s choice of pharmacy. The patient will then be responsible for obtaining the medication from the pharmacy.

Based on clinical assessments and need, and following standard clinical guidelines for MOUD [43], clinicians will also offer OUD treatment options that may include: buprenorphine/naloxone, adherence counseling/support for methadone, or coordination with outpatient treatment providers. The specific PrEP and MOUD regimen (doses and/or combination of medications) is not dictated by the protocol and will be instead up to the discretion of the clinician and patient preference. Clinicians are not required by the protocol to prescribe PrEP. PrEP provision is based on patient preference and clinician judgment; it is possible that participants will not be prescribed PrEP or MOUD during the clinical visit. Even if participants are not prescribed PrEP and/or MOUD, they will remain in the study and continue with follow-up study visits.

Resource availability will likely vary depending on context (CT vs. AL), which is one of the reasons we are stratifying by site and will evaluate site differences. A dummy code will be used for billing, so there will be no provider charge to the participant for the encounter. Clinicians will electronically order refills and send PrEP/MOUD prescriptions to the patient’s pharmacy, which will be billable to the patient’s insurance. Follow-up laboratory tests will also be ordered, and billing will be site-dependent. Following the visit, the clinician will document the encounter in the EHR and complete the post-visit checklist in REDCap for quality assurance and to ensure intervention fidelity. We will comply with all federal and state regulations regarding buprenorphine prescribing using telehealth [44, 45]. Procedures will be modified as needed to accommodate new regulations and laws as they emerge.

#### Enhanced standard of care arm

Decision aid-only participants will receive a printed document that verifies participation in a research study, results from POC testing and confirmation of PrEP eligibility, and a tailored list of area PrEP providers from the AIDSVu PrEP Locator, based on insurance status, location, and scheduling preferences [46]. We will pre-screen the PrEP locator output to ensure that clinics are open to care and accepting new patients, but will not provide PrEP navigation. In contrast to treatment as usual (wherein few WICL can access PrEP), this arm involves an individualized decision aid and onsite POC HIV testing with confirmation of PrEP eligibility in addition to personalized referral services.

#### Follow-up

**Fig. 1:**
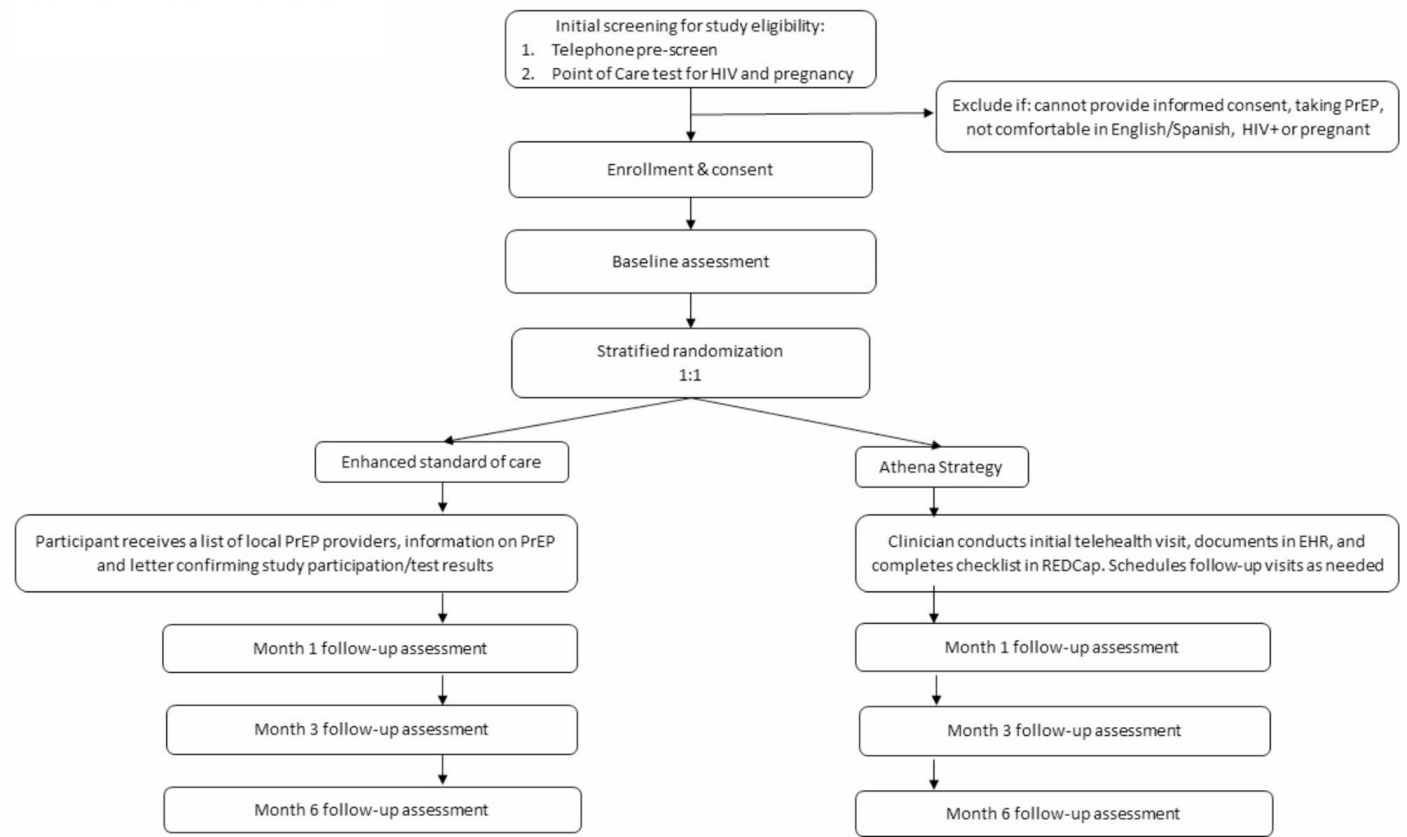
Timeline of study activities

Following the baseline interview, participants will be followed with study interviews at months 1, 3, and 6, according to the participant timeline (Fig. 1). Participants will receive a text message or phone call reminder of appointments two days prior to each interview, and researchers will make up to 3 attempts to reschedule missed appointments before marking as “lost to follow up.”

Participants who become incarcerated during the 6-month observation period will continue to be followed and can complete study interviews in-person while in jail or prison, or on return to the community. Participants on PrEP or MOUD may still receive clinical care and follow-up during the incarceration period, though it depends on the discretion of the prison or jail-based clinical providers. All procedures will be approved by the relevant review boards at each CL site prior to study activities, and research staff will obtain necessary approvals to enter CL facilities. Participants who complete study interviews during incarceration may retrieve participation compensation upon release, per CL site regulations. Participants incarcerated > 6 months will be disenrolled and invited to re-enroll if they meet eligibility requirements upon their return to communities. We will re-consent them upon their return to communities to ensure they do not experience real or perceived coercion.

For participants in either arm who receive PrEP and/or MOUD during the study, the prescribing clinician (including the prescribing clinician for Athena participants if applicable) will be responsible for monitoring and managing potential side effects of PrEP and/or MOUD. To minimize missed appointments, clinicians will assess any possible barriers to completing the appointment (i.e., transportation or connectivity issues) and send a reminder call or text message the day before the appointment. Alternatively, participants may request to schedule their follow-up clinician visit on the same day as their follow-up interview for convenience. If participants are identified as having a new health condition (for example, a new diagnosis of an STI, pregnancy, HIV, or viral hepatitis), we will adhere to institutional and treatment guidelines and provide referrals for treatment and care. Participants testing positive for HIV or pregnancy on follow-up will be excluded from further study participation and referred to appropriate services.

### Measures

As shown in the Supplementary Table, the primary outcome of the study is PrEP initiation, which we measure by pharmacy fill date (record review) with a signed release of information. The secondary outcome is PrEP adherence by objective measure, which we assess with a urine assay for tenofovir levels (for participants initiating tenofovir-based PrEP) and injection dates for patients initiating injectable cabotegravir for PrEP. Additional health behavior outcomes of interest are shown in the Table, including self-reported PrEP adherence, 6-month PrEP care retention, use of other HIV prevention tools, engagement in the OUD treatment cascade, and new diagnoses of HIV, viral hepatitis, STIs, or pregnancy. We will review the EHR medication list for PrEP and/or MOUD prescriptions after each clinical visit. If patients receive PrEP and/or MOUD prescriptions outside of our respective EHR-covered health systems, the research assistant will obtain prescription fill and refill dates from clinics and pharmacies with a signed release of information and a phone call on each follow-up interview day.

#### Implementation outcomes

We will conduct focus groups with participants (as consumers) and healthcare providers in a community advisory board (as setting-level participants) We will use the implementation framework and taxonomy defined by Proctor et al. [38] to guide our evaluation of the relative advantage of Athena over the decision aid alone from the perspective of stakeholders at multiple levels (patients, healthcare systems, system administrators, policy-makers). The taxonomy of implementation outcomes and their applied definition are shown in the Table 1.

**Table 1:** Taxonomy of implementation outcomes

		Level of analysis (measurement)		
**Outcome**	**Definition**	**Consumer level**	**Healthcare provider level**	**Setting level**
Acceptability	Stakeholder perception that Athena is acceptable	What needs to change in Athena design and delivery?	What needs to change in Athena design and delivery?	Acceptability of Athena delivery of integrated PrEP/MOUD
Adoption	Intention to employ Athena in practice	Interaction time/frequency and quality	Barriers/facilitators to staff using Athena for WICL in each setting	Barriers/facilitators to using Athena for WICL in each setting
Feasibility	Extent to which Athena can be successfully used in settings serving WICL	Logistical constraints and structural barriers to Athena	Logistical constraints and structural barriers to Athena	Logistical constraints and structural barriers to Athena
Fidelity	Degree to which Athena was implemented as planned	Was Athena delivered as intended?	Was Athena delivered as intended? Athena clinicians will complete pre-session checklists and post-session quality assurance forms	
Implementation cost	Cost of implementing Athena to promote sustainability	Social (stigma), economic cost of participation in Athena	Time, staffing cost of providing Athena	Economic cost of delivering Athena (e.g., reimbursement concerns)
Penetration/Reach	Integration of Athena within settings serving WICL	Recruitment rate; Completion rate; reasons for nonparticipation	Clinician agreement to recommend Athena in settings serving WICL	Administrators’ agreement to employ Athena in settings serving WICL
Sustainability potential	Extent to which Athena becomes institutionalized or is used continuously after study completion in settings serving WICL	What are barriers to Athena continuation after study completion? What would need to change to continue Athena?	What gets in the way of incorporating Athena into current clinical practice and what would need to change?	What are the barriers to scaling up and out Athena beyond the current study? Sustainment measurement system scale

WICL = women in the criminal legal system

MOUD = medications for opioid use disorder

### Data monitoring

A Data Safety Monitoring Board is in place to monitor study progress, and is comprised of experts in criminal legal systems, HIV prevention, and clinical trial methods. A DSMB Charter has been finalized and adopted and is available upon request. DSMB meetings are held a minimum of every 12 months and interim data analyses will occur if and when recommended by the DSMB.

## Statistical analyses

### Sample size

The study is powered on the primary outcome, PrEP initiation. The null hypothesis (H0) is that the proportion of participants initiating PrEP in the Athena arm will be the same as in the decision aid-only arm, whereas the alternative hypothesis (H1) is that the proportion of participants initiating PrEP in the Athena arm will be higher than those in the decision aid-only arm. Based on our clinical trial of a decision aid [33], we anticipate ~ 20% of decision aid-only participants will achieve the primary outcome. Based on our demonstration project of eHealth for PrEP for WICL [36], we anticipate 40–50% of Athena participants will achieve the primary outcome. A total sample size of 212 (106 in each group) would achieve 90% power to detect a 20% absolute difference between the two arms. The proportion in the Athena arm is assumed to be 20% under the null hypothesis and 40% under the alternative hypothesis. The proportion in the decision aid-only arm is < 30%. We will inflate the sample size by 15% to account for attrition and will plan to enroll 250 participants total across 2 sites, which is highly feasible. Though we attempt to minimize missing data by having an objectively measured outcome, we have considered additional plans for missing data that include performing both intention-to-treat and per protocol analyses and performing multiple imputation sensitivity analyses if appropriate.

### Primary analyses

We will evaluate the effect of the Athena strategy, as compared to the decision aid-only (active control) in terms of the primary outcome, PrEP initiation. We will first conduct a descriptive analysis to characterize the study sample in terms of baseline characteristics overall, by treatment arm, and by site. The primary outcome (proportion of participants initiating PrEP) will be compared by treatment arm, site, and race/ethnicity using chi-square techniques. Using an intention-to-treat approach, we will build a multivariate logistic regression model of PrEP initiation; participant characteristics will first be evaluated in univariate models and, if significant at a more liberal p-value threshold, will be used in the multivariate model. In a per-protocol analysis, we will exclude time periods during which individuals are incarcerated during study follow-up, and thus removed from community exposure. The log-rank chi-square test (Kaplan-Meier survival analysis) will be used to compare time to first PrEP visit and time to PrEP initiation between the two arms. We will use descriptive analyses to characterize the PrEP care continuum for the entire sample over 12 months of follow-up, stratified by randomization arm and comprising the following steps [29]: HIV risk, PrEP awareness, completed PrEP visit encounter, PrEP initiation, PrEP adherence, and PrEP care retention by 1-, 3-, and 6-months post-baseline. We will describe engagement in the OUD treatment cascade both overall and by study arm, in terms of treatment initiation, retention, and substance use remission. Participant profiles will be created based on baseline characteristics (within each treatment arm) to assess changes in outcomes over time by site, study arm, and site by study arm. Depending on the distribution of such profiles, we will conduct a latent class analysis to explore characteristics of individuals as they relate to the established profiles. We will assess substance use over time with repeated measures and model using generalized estimating equations (GEE), by study arm and by site. We will assess for the complexity of SUD treatment (e.g., court-mandated vs. voluntary, behavioral vs. MOUD) and explore changes in treatment engagement over time, depending on substance use treatment modality. We will similarly describe diagnosis and treatment engagement for comorbid Hepatitis C and sexually transmitted infections over the 6-month period of observation.

### Secondary analyses

We will model individual- and population-level effects on averted HIV infections and quality-adjusted life-years (QALY); then we will use qualitative analysis guided by the Proctor Framework to understand process measures important to potential Athena scale-up [38]. For the modeling component of the study, we will employ state transition Markov modeling techniques to estimate long-term outcomes in terms of averted HIV infections and QALY gains at the individual level. Markov models offer a powerful tool to simulate individual transitions through a set of mutually exclusive states over time [47]. The model will include states characterized by HIV status and PrEP enrollment, as well as MOUD enrollment. Health utility weights of different health states will be estimated from literature [48]. Population-level effects from scaling up PrEP using the Athena strategy and decision aid-only will be evaluated using compartmental dynamic transmission modeling techniques [49]. We will stratify a hypothetical population by their behavioral characteristics predictive of HIV transmission risk and run the model assuming different intervention scale-up levels. In the base case analysis, we will run the models over a 10-year time horizon and will explore the sensitivity to this timeframe by running the models over 5- and 15-year horizon. Descriptive statistics will be used to evaluate structured survey data. For the nominal group technique (NGT) component, as we have done previously [50–54], we will tabulate votes to immediately rank priorities. Once group ranking is completed, we will undertake a thorough discussion to ensure the ranking has face validity to hear minority voice perspectives. If there is lack of clarity in the NGT ranking and for in-depth interviews, the transcript will be reviewed by two independent reviewers to decide if the responses are convergent or divergent, and where there is disagreement a third person will break the tie [55, 56]. NGT and interview transcripts will be analyzed separately for each purpose in NVivo based on modified grounded theory methods [57] and thematic content analysis [58] involving an iterative process of reading transcripts, identifying themes, and forming a coding scheme. To imbue sense into the thematic codes, analysis will be guided by the Proctor implementation outcomes framework [38]. Context will be an important consideration at all levels of the framework, and we will specifically assess for key site differences in organizational/process outcomes from these multi-level perspectives because similarities and differences by site are important for implementation and to ensure there is generalizable knowledge for future scale-up.

Once the findings are compiled, to move from evaluation to action, we will convene a stakeholder policy symposium at each site to present findings and discuss incorporation of findings into Athena sustainability plans in each state. Findings will be presented to community advisory boards at each site at the end of the study to identify opportunities to advocate for systems change. We will invite policy-maker participants (e.g., state health officers, state Medicaid policy-makers) to join the investigative team to produce a white paper on policy issues related to the Athena strategy of eHealth delivery of integrated PrEP/MOUD.

## Discussion

This study uses eHealth to deliver integrated PrEP and MOUD to a population of WICL with OUD who are in high need of accessible prevention and treatment services that meet them where they are. The project is being implemented in two very different contexts- New Haven, CT and Birmingham, AL- which is important to inform future scaling-up and scaling-out of this intervention strategy.

While prior research has confirmed the need for and interest in PrEP among WICL [59–62], the linkage step in the PrEP care continuum is especially challenging for this patient population [29], who experience individual (mistrust, stigma, trauma, reduced health literacy) and structural (transportation, childcare, cost) barriers to care and challenges navigating siloed healthcare systems [33, 63, 64]. Existing traditional brick and mortar models of PrEP delivery are insufficient to meet women’s needs, and they are separated from SUD treatment. Because of limitations to funding streams for reimbursement, staffing, and resources, dedicated Ryan White-funded PrEP clinics often do not provide MOUD, and MOUD clinics rarely offer PrEP. Decades of research has demonstrated that, for people living with HIV, integration of MOUD with HIV treatment and care is beneficial in terms of HIV and SUD outcomes [65, 66]. Now low-barrier delivery models are needed for integration of PrEP and MOUD, which reflects a holistic approach to patient-centered care.

Electronic health (eHealth) is a powerful tool that can overcome many challenges to integrated PrEP/MOUD delivery to WICL by reducing stigmatizing face-to-face encounters with clinicians and overcoming geographic constraints [16, 67, 68]. Telehealth, a component of eHealth [69], has been used to manage chronic health conditions in physician shortage areas and is endorsed by the Infectious Disease Society of America [70] for specialty care with emerging models for its use in PrEP delivery. A systematic review of existing telePrEP programs found they were highly feasible, acceptable, and effective at expanding the reach of PrEP [71]. Existing research-based and commercial telePrEP programs nearly exclusively focus on non-Hispanic white privately insured men who have sex with men, limiting relevance to WICL. Prior telePrEP programs that have direct patient-provider contact also primarily involve synchronous videoconferencing, which limits potential application to WICL who lack adequate technological support [71–73]. In contrast, eHealth programs leverage multiple modes of electronic communication, including phone or text-based support, e-prescribing, electronic lab ordering and reporting with electronic health records (EHR) to facilitate coordination of care between patients, providers and health systems. eHealth can be cost-effective, engage patients, and reach stigmatized and marginalized populations like WICL with OUD [74–78]. eHealth has emerged as a powerful strategy during the disruptions of COVID-19, by reducing individuals’ discomfort and distrust of disclosing risk behaviors, providers’ low cultural competency for working with individuals of diverse identities and with substance use, and bypassing barriers to healthcare for marginalized populations [79–83], all features crucial for WICL. It can further guide prevention delivery and health decision-making in a confidential, less stigmatizing, and convenient manner [84–90]. 

To adequately scale eHealth for PrEP in the U.S., we must consider the local adoption environment, where current public and private insurance policies, reimbursement, and payment structures are obstacles to care delivery [78]. For example, in both CT and AL, live interactive videoconferencing is required for “face-to-face interaction”—phone, e-mail, or texting interactions are not generally reimbursed. This is especially problematic for WICL who may lack sufficient data plans or broadband coverage for live videoconferencing and need other methods of communication [71–73]. Prior to COVID-19, patients had to be located at an “established medical site” for reimbursement and, while these sites may be more convenient [91], WICL may still face stigma and shame when disclosing HIV risk behaviors, which is limiting for care engagement. In states that did not expand Medicaid under the Affordable Care Act (like AL), un- and under-insurance present other challenges to PrEP access. While “Ready Set PrEP” and drug assistance programs can reduce the need for copays, patients still need to cover the cost of provider visits and laboratory testing that may be prohibitive.

There are further legal and policy hurdles to using eHealth for delivery of MOUD. The public health emergency period during the height of the COVID-19 pandemic paused the federal requirement for an in-person visit prior to initiation of controlled substances, which was enabling for access to MOUD (methadone is a Schedule II and buprenorphine a Schedule III narcotic.) Though the public health emergency period ended in May 2023, this flexibility to controlled substance prescribing via telehealth was extended through November 2023 (and as long as November 2024 if a patient-provider relationship was already established.) [92] The Drug Enforcement Agency has announced proposed permanent rules for prescribing controlled substances using telemedicine that could further limit access to buprenorphine, pending public comment [93]. In the interim, some state laws are significantly more restrictive. For example, in Alabama, a passed Senate Bill requires that “the prescriber has had at least one in-person encounter with the patient within the preceding 12 months.” [94].

For patients with highest need for MOUD, including WICL, telehealth is a stigma-reducing intervention that reduces barriers to entry. Telehealth is healthcare. The study described here will foster understanding of how to best navigate legal and policy limitations to telehealth access for MOUD and to integrate it with PrEP. In closing, beyond a sustainable business model for eHealth for PrEP, implementation science can consider how best to use eHealth to deliver integrated PrEP/MOUD as an evidence-based practice in diverse micro-epidemics, which is especially crucial for women involved in criminal legal systems.

## Electronic supplementary material

Below is the link to the electronic supplementary material.

Supplementary Material 1

Supplementary Material 2

## Data Availability

The study is registered on Clinicaltrials.gov (NCT05547048). Additional materials available on request to the contact author.

## Abbreviations

CL: criminal legal
PrEP: pre-exposure prophylaxis
MOUD: medications for opioid use disorder
OUD: opioid use disorder
IPV: Intimate partner violence
IRB: Institutional Review Board
POC: point-of-care
SOC: standard of care
STI: sexually transmitted infection
SUD: Substance use disorder
WICL: Women involved in criminal legal systems
