# Supplementary material for: Rationale and design of a randomized clinical trial of integrated eHealth for PrEP and medications for opioid use disorders for women in the criminal legal system. The Athena study
Source: Addict Sci Clin Pract. 2025 Jan 17;20:4. doi: 10.1186/s13722-024-00534-x (PMC11742507; doi:10.1186/s13722-024-00534-x)
Supplement: Supplementary file 2 — Supplementary Material 2 [file 13722_2024_534_MOESM2_ESM.docx]

**Supplementary Table. Operationalization and Rationale for Study Measures**

Participants in both arms will be assessed with study visits over 6 months (see Table). Letters in the table refer to the source of data collection (I, P, T, R). Data will be derived from study interviews (I), participant-entered data from the decision aid (P), POC testing (T) and EHR record review (R) as shown. For structured interview (I) visits, RAs can conduct them remotely (via phone) or in person for participants in both arms to minimize reporting bias. Structured interviews will be conducted at months 0, 1, 3 and 6 after randomization. As noted in the Table below, the only laboratory testing (T) in follow-up for participants in both arms is collection of urine for toxicology and tenofovir level testing. Data from all sources will be entered into RedCAP by the RA. We use a combination of interviews and other collected data to reduce participant burden. Though we acknowledge the potential for artifact given the frequency of follow-up, we are engaging with women in service settings that they already attend with much higher frequency, for example weekly community supervision or syringe exchange; procedures are intentionally convenient and congruent with existing behaviors.

| Area to assess aligned with the Behavioral Health Framework | Operationalization and Rationale | Months since randomization  0 1 3 6 | | | |
| --- | --- | --- | --- | --- | --- |
| *Predisposing characteristics* | | | | | |
| Demographic characteristics | age, race/ethnicity, gender identity, sexual orientation, zip code | I |  |  |  |
| Structural violence and CJ involvement | lifetime arrests, lifetime incarcerations, time since last incarceration, duration last incarceration, charged offenses | I | I | I | I |
| Healthcare status | past medical history, recent hospitalizations, current medications, health care utilization, | I | I | I | I |
| *Enabling/disabling factors* | | | | | |
| Violence/victimization exposure | Physical IPV: Revised Conflicts Tactics Scale-2(1, 2)  Sexual IPV: Sexual Experiences Survey.(3)  Psychological IPV: Psychological Maltreatment of Women Inventory.(4) | I |  |  |  |
| Modifiable demographics | housing status, employment, income, health insurance (and primary holder) | I | I | I | I |
| Barriers to treatment | Allen barriers to treatment survey for women(5) | I |  |  |  |
| Self-efficacy | General self-efficacy scale(6) | I | I | I | I |
| Resilience | Connor-Davidson RISC score (25 items), 5 key resilience domains(7) | I |  |  |  |
| Telehealth readiness | Validated assessment of patient readiness, engagement, health literacy (8) | I |  |  |  |
| PrEP stigma | PrEP stigma scale(9) (replicated with permission from lead author) | I | I | I | I |
| PrEP readiness | Validated 25-item instrument on readiness for HIV treatment with ART(10) that has been adapted for PrEP in women(11) | I |  |  |  |
| PrEP preference | Pre- and post-grid single-item assessments of decisional preference for and interest in PrEP on a 5-point Likert scale.(12, 13) | P |  |  |  |
| HIV risk perception | Perceived personal and partners’ HIV risk (2-items Likert scale)—repeated measure as prior PrEP clinical trials of MSM have demonstrated decreased risk perception as a motivation for stopping PrEP.(14) | P | I | I | I |
| Religiousness/spirituality | Brief Multidimensional Measure of Spirituality:(15) documented key social determinant of health in Black women in the South(16) | I |  |  |  |
| Social support | RAND Social Support Scale:(17) encompasses emotional/informational, tangible, affectionate support and positive social interactions | I |  |  |  |
| *Need factors* | | | | | |
| HIV risk behaviors | NIDA’s modified risk behavior assessment:(18) measured repeatedly to assess for potential risk compensation. We also want to measure whether HIV risk behaviors decrease because of the intervention. | P | I | I | I |
| Partner-level HIV risk factors | Partner recent incarceration and partner concurrency(19) demonstrated to reflect HIV risk among Black women | P |  |  |  |
| Depression severity | Center for Epidemiologic Studies Depression Scale (CES-D)(20) standardized screening and diagnosis for depressive symptoms because there is a high degree of depression in this population | I |  |  |  |
| Substance use disorder (SUD) severity | DAST-10(21, 22) and NIDA-modified ASSIST(23, 24)  AUDIT-C(25) for alcohol use disorder | I |  |  |  |
| Ongoing substance use | Urine toxicology screen: To be convenient and patient-centered and lower barriers to access to PrEP, participants completing study interviews virtually can collect urine in sterile containers and store them at Quest for later retrieval and testing by RAs. There are no special storage requirements for these specimens. | T |  | T | T |
| Reproductive health needs | Lifetime sexual and reproductive health needs assessment that we have developed and used in prior PrEP clinical trials(26) | I |  |  |  |
| *Health Behavior Outcomes* | | | | | |
| *Primary:* PrEP initiation | Pharmacy fill date to reduce potential recall or ascertainment bias |  | R | R | R |
| *Secondary:* PrEP adherence by objective measure (48-hour perspective) | Urine assay for tenofovir levels (single point of care qualitative immunoassay), which we selected over plasma-based measures because of greater sensitivity, lower cost, faster turnaround time (enabling real-time feedback) and no special processing requirements(27) |  |  | T | T |
| PrEP adherence by self-report (30-day perspective) | A new 3-item self-report adherence measure with excellent psychometric characteristics and construct validity.(28) We will measure adherence multiple ways to generate the most complete picture of PrEP as recommended.(29) |  | I | I | I |
| 6-month PrEP care retention | Dates of completed PrEP encounters in EHR and mechanism of encounter (i.e., clinic dates, eHealth delivery for Athena arm). 6-month retention was selected based on national data of Medicaid-insured patients: median PrEP persistence is 5.8 months (95%CI 4.1-7.1) in women; 4.7 months (95%CI 4.0-6.2) in people who are Black.(30) |  | R | R | R |
| Use of other HIV prevention tools | Male or female condom use, syringe service programs, voluntary reduction of sex partners, U=U for women with HIV+ male partners. | P | I | I | I |
| OUD treatment cascade engagement | The OUD treatment cascade is defined by: 1) initiation of MOUD (or other evidence-based treatment); 2) 6-month retention on treatment; and 3) remission.(31, 32) We will assess for complexity of SUD treatment (e.g., court-mandated vs. voluntary, behavioral vs. MOUD, residential vs. outpatient, detoxification program, etc.) | I | I | I | I |
| HIV, viral hepatitis, STI, or pregnancy diagnosis | Dates and results of guideline-driven follow-up testing quarterly. | T |  | R | R |

1. Straus M, Hamby S, Boney-McCoy S, Sugarman D. The Revised Conflict Tactics Scales (CTS2): Development and Preliminary Psychometric Data. Journal of Family Issues. 1996.;17(3):283-316.

2. Roberts ST, Haberer J, Celum C, Mugo N, Ware NC, Cohen CR, et al. Intimate Partner Violence and Adherence to HIV Pre-exposure Prophylaxis (PrEP) in African Women in HIV Serodiscordant Relationships: A Prospective Cohort Study. J Acquir Immune Defic Syndr. 2016;73(3):313-22.

3. Koss MP, Gidycz CA, Wisniewski N. The Scope of Rape - Incidence and Prevalence of Sexual Aggression and Victimization in a National Sample of Higher-Education Students. Journal of Consulting and Clinical Psychology. 1987;55(2):162-70.

4. Tolman RM. koss. Violence Vict. 1999;14(1):25-37.

5. Allen K, Dixon M. Psychometric assessment of the Allen Barriers to Treatment Instrument. The International journal of the addictions. 1994;29(5):545-63.

6. Schwarzer R, Jerusalemn M. Generalized Self-Efficacy Scale. In: Weinman J, Wright S, Johnston M, editors. Measures in health psychology: A user’s portfolio Causal and control beliefs. Windsor, UK: NFER-NELSON; 1995. p. 35-7.

7. Connor KM, Davidson JR. Development of a new resilience scale: the Connor-Davidson Resilience Scale (CD-RISC). Depress Anxiety. 2003;18(2):76-82.

8. Maryland Health Care Commission. Telehealth Readiness Assessment Tool 2019 [Available from: <https://mhcc.maryland.gov/mhcc/pages/hit/hit_telemedicine/documents/TLHT_TRA_Tool.pdf>.

9. Calabrese SK, Dovidio JF, Tekeste M, Taggart T, Galvao RW, Safon CB, et al. HIV Pre-Exposure Prophylaxis Stigma as a Multidimensional Barrier to Uptake Among Women Who Attend Planned Parenthood. J Acquir Immune Defic Syndr. 2018;79(1):46-53.

10. Fernandez MI, Hosek S, Warren JC, Jacobs RJ, Hernandez N, Martinez J. Development of an easy to use tool to assess HIV treatment readiness in adolescent clinical care settings. AIDS Care. 2011;23(11):1492-9.

11. Celum C, Delany-Moretlwe S, Hosek S, Dye B, Bekker LG, Mgodi N, et al. Abstract #1049: Risk Behavior, Perception, and Reasons for PrEP among Young African Women in HPTN 082. Conference on Retroviruses and Opportunistic Infections (CROI); Boston, MA2018.

12. The Ottawa Hospital Research Institute. Patient Decision Aids 2016 [Available from: <https://decisionaid.ohri.ca/AZsearch.php?criteria=hiv&search=Go>.

13. Brehaut JC, O'Connor AM, Wood TJ, Hack TF, Siminoff L, Gordon E, et al. Validation of a decision regret scale. Medical decision making : an international journal of the Society for Medical Decision Making. 2003;23(4):281-92.

14. Zimmermann HM, Eekman SW, Achterbergh RC, Schim van der Loeff MF, Prins M, de Vries HJ, et al. Motives for choosing, switching and stopping daily or event-driven pre-exposure prophylaxis - a qualitative analysis. J Int AIDS Soc. 2019;22(10):e25389-e.

15. Johnstone B, Yoon DP, Franklin KL, Schopp L, Hinkebein J. Re-conceptualizing the Factor Structure of the Brief Multidimensional Measure of Religiousness/Spirituality. Journal of Religion and Health. 2008;48(2):146.

16. Nunn A, Jeffries WL, Foster P, McCoy K, Sutten-Coats C, Willie TC, et al. Reducing the African American HIV Disease Burden in the Deep South: Addressing the Role of Faith and Spirituality. AIDS and Behavior. 2019;23(3):319-30.

17. Sherbourne C, Stewart A. The MOS Social Support Survey. Santa Monica, CA: RAND corporation; 1993.

18. Fisher JD, Fisher WA, Cornman DH, Amico RK, Bryan A, Friedland GH. Clinician-delivered intervention during routine clinical care reduces unprotected sexual behavior among HIV-infected patients. J Acquir Immune Defic Syndr. 2006;41(1):44-52.

19. Sales JM, Sheth AN. Associations Among Perceived HIV Risk, Behavioral Risk and Interest in PrEP Among Black Women in the Southern US. AIDS and behavior. 2019;23(7):1871-6.

20. Radloff LS. The CES-D scale: A self report depression scale for research in the general population. . Applied Psychological Measurement. 1977;1:385-401.

21. Skinner HA. Drug Abuse Screening Test-10 Toronto, Canada: Centre for Addiction and Mental Health; 1982 [Available from: <http://www.bu.edu/bniart/files/2012/04/DAST-10_Institute.pdf>.

22. Yudko E, Lozhkina O, Fouts A. A comprehensive review of the psychometric properties of the Drug Abuse Screening Test. J Subst Abuse Treat. 2007;32(2):189-98.

23. World Health Organization. The Alcohol, Smoking and Substance Involvement Screening Test (ASSIST): development, reliability and feasibility. Addiction. 2002;97(9):1183-94.

24. National institute on Drug Abuse. Resource Guide: Screening for Drug Use in General Medical Settings (The NIDA Quick Screen and NIDA-modified ASSIST) 2019 [Available from: <https://www.drugabuse.gov/publications/resource-guide-screening-drug-use-in-general-medical-settings/nida-quick-screen>.

25. Reinert D, Allen J. The Alcohol Use Disorders Identification Test (AUDIT): a review of recent research. Alcohol Clin Exp Res. 2002;26(2):272-9.

26. Hoff E, Adams ZM, Grimshaw A, Goddard-Eckrich DA, Dasgupta A, Sheth SS, et al. Reproductive Life Goals: A Systematic Review of Pregnancy Planning Intentions, Needs, and Interventions Among Women Involved in US Criminal Justice Systems. J Womens Health (Larchmt). 2020.

27. Koenig HC, Mounzer K, Daughtridge GW, Sloan CE, Lalley-Chareczko L, Moorthy GS, et al. Urine assay for tenofovir to monitor adherence in real time to tenofovir disoproxil fumarate/emtricitabine as pre-exposure prophylaxis. HIV Med. 2017;18(6):412-8.

28. Wilson IB, Lee Y, Michaud J, Fowler FJ, Jr., Rogers WH. Validation of a New Three-Item Self-Report Measure for Medication Adherence. AIDS Behav. 2016;20(11):2700-8.

29. Agot K, Taylor D, Corneli AL, Wang M, Ambia J, Kashuba ADM, et al. Accuracy of Self-Report and Pill-Count Measures of Adherence in the FEM-PrEP Clinical Trial: Implications for Future HIV-Prevention Trials. AIDS and behavior. 2015;19(5):743-51.

30. Huang YA, Tao G, Smith DK, Hoover KW. Persistence With Human Immunodeficiency Virus Pre-exposure Prophylaxis in the United States, 2012-2017. Clin Infect Dis. 2021;72(3):379-85.

31. Williams AR, Nunes EV, Bisaga A, Pincus HA, Johnson KA, Campbell AN, et al. Developing an opioid use disorder treatment cascade: A review of quality measures. Journal of substance abuse treatment. 2018;91:57-68.

32. Socías ME, Volkow N, Wood E. Adopting the 'cascade of care' framework: an opportunity to close the implementation gap in addiction care? Addiction (Abingdon, England). 2016;111(12):2079-81.
